# Preferences, Perceptions, and Use of Online Nutrition Content Among Young Australian Adults: Qualitative Study

**DOI:** 10.2196/67640

**Published:** 2025-09-29

**Authors:** Bill Tiger Lam, Ewa A Szymlek-Gay, Christel Larsson, Claire Margerison

**Affiliations:** 1 School of Exercise and Nutrition Sciences Institute for Physical Activity and Nutrition Deakin University Geelong Australia; 2 Department of Food and Nutrition, and Sport Science University of Gothenburg Gothenburg Sweden

**Keywords:** online nutrition content, young adults, social media, internet, qualitative, perceptions, preferences, nutrition, online, Australia, misinformation

## Abstract

**Background:**

Nutrition misinformation is pervasive on frequently accessed online sources such as social media platforms and websites. Young adults are at a high risk of viewing or engaging with this content due to their high internet and social media usage.

**Objective:**

This study aimed to understand young adults’ preferences, perceptions, and use of online nutrition content.

**Methods:**

Young Australian adults (aged 18-25 years) were recruited and interviewed individually via video calling (Zoom; Zoom Video Communications) between December 2023 and February 2024. Participants were recruited via convenience sampling using Facebook advertising. The interviewer followed a semistructured format, and questions were guided using a piloted template. Reflexive thematic analysis was conducted using NVivo (Lumivero) to explore the preferences, perceptions, and use of online nutrition content among the sample.

**Results:**

The sample (N=20; mean age 22.9 y, SD 2.3 y) was predominantly female (n=13, 65%) and had, or was studying toward, a tertiary qualification (16/17, 94%). Most participants used social media (19/20, 95%) and internet websites (16/20, 80%) to access nutrition content. Other platforms used included generative artificial intelligence (n=1), apps (n=1), eBooks (n=1), newsletters (n=1), and podcasts (n=1). When exploring perceptions, most participants agreed that online nutrition content was quick and easy to find and informative. Furthermore, perceived reliability and engagement depended on several factors such as the creator’s credentials, length and format of content, consensus on topics, and sponsorships. Short-form content was not considered reliable, despite its engaging nature. Content containing sponsorships or product endorsements was met with skepticism. However, participants were more likely to trust content reportedly created by health professionals, but it was unknown whether they were accessing verified professionals. The oversaturation of content demotivated participants from evaluating the reliability of content. When asked about preferences, participants valued both short- and long-form content, and evidence-based content such as statistics and references and preferred casual and entertaining content that incorporated high-quality and dynamic editing techniques such as voiceovers.

**Conclusions:**

The study identified the online nutrition content sources and topics young Australian adults access and the key factors that influence their perceptions and preferences. Young Australian adults acknowledge that misinformation is not exclusive to certain platforms. The accessibility and engagement of content and the ambiguity of professional “credentials” may lead them to trust information that is potentially of low quality and accuracy. Findings also show that there needs to be a balance between engaging formats and presenting evidence-based information when designing online nutrition content to engage these audiences while combatting nutrition misinformation. Future research should explore how these factors impact usage of online nutrition content and dietary behaviors among young Australian adults. Further consultation with this cohort can inform tailored interventions that aim to enhance their food and nutrition literacy and diet quality.

## Introduction

The pervasiveness and hyper-accessibility of online health and nutrition content worldwide enable the proliferation of misinformation in growing online environments. Research indicates that the majority of health and nutrition information on the internet and social media is of poor quality and accuracy [1-3]. The quality of online nutrition content is evaluated against a number of criteria [4], such as accuracy, defined as the factual correctness of information indicated from scientific literature or published guidelines [1], and credibility, which includes disclosure of sponsorships and references. Considering the growing use of the internet [5] and the volume of content accessible, there is concern regarding the negative impact of misinformation on population health [6].

The use of online nutrition content in Australia has increased significantly over the past years [7] in line with the growing amount of nutrition-related content posted online. Online nutrition content in the Australian context refers to any material, for example, text, image, video, or audio, discussing food and nutrition-related topics that is shared online. Evidence shows that the internet is currently the most frequently used source for nutrition information, compared to traditional sources, for example, magazines and television, and professional sources, for example, dietitians and nutritionists [8]. Although professional sources are regarded as reliable and effective by consumers, the ease, inexpensiveness, and immediacy of online sources are strong influences to use online sources over those of nutrition professionals [9,10]. Coupled with the growing prevalence of misinformation online [6] about health and nutrition, accessing nutrition content online may lead to harmful effects on the health, well-being, and economic status of individuals [2,11,12].

In particular, online nutrition content can have a large impact on young adults’ (aged 18-25 years old) dietary choices [13] as they characterize a large proportion of consumers who access the internet and social media [14,15] and are more likely to engage with this content compared to middle-aged and older adults [7,10,16]. Research indicates that young adults also have the poorest diet quality compared to other age groups, which may put them at risk of nutrient deficiencies or overnutrition [17]. Therefore, there is a need to identify the online sources young adults use for nutrition content and how they perceive these. In addition to identifying use and perceptions, exploring young adults’ preferences for nutrition content is also needed. Exploring how young adults use the internet for nutrition content and what topics they engage with can identify opportunities for targeted intervention. In addition, understanding their perceptions of the reliability and engagement of online sources and their preferences can guide health professionals in creating effective evidence-based content. However, there is a scarcity of literature that investigates these areas [13,18]. Therefore, by using a qualitative approach, this study aimed to characterize the use of online nutrition content among young Australian adults, explore how they perceive the content, and identify their preferred characteristics of content.

## Methods

### Study Design

A constructivist grounded theory approach was used to explore young Australian adults’ use, preferences, and perceptions of online nutrition content. This methodological approach involves the generation of a theory that has been grounded in the coded data. This enables richer and deeper exploration beyond observation of behaviors, and therefore results in nuanced understandings of what, how, and why such behaviors occur [19]. The consolidated criteria for reporting qualitative research checklist [20] was used as a guideline for the conduct and reporting of this paper (see Multimedia Appendix 1).

### Participants and Recruitment

Eligible participants included young adults (aged 18-25 years) residing in Australia. Exclusion criteria included an inability to verbally communicate in English or an inability to engage with nutrition information or content online.

Participants were recruited between December 2023 and February 2024 via convenience sampling using social media advertisements (Facebook; Meta Platforms). The link on the advertisements took the participants to a Qualtrics survey to confirm their eligibility. If the participant was eligible, they were asked to provide their availability and an email address. This information was used to contact the participant in the study and to schedule an interview via email.

The study aimed to recruit between 20 and 25 participants. This was set as theoretical data saturation in similar studies was reached at 21-22 interviews [21,22]. Interviews stopped when the interviewer declared data saturation was reached, that is, no new valuable codes were extracted at the last interview, which was confirmed during data analysis.

### Data Collection

Interviews were conducted using a semistructured format. Open-ended questions with additional probing questions were developed to elicit information on the use of online nutrition content, perceptions of content, and preferred characteristics of content. The interview questions involved asking participants about what online platforms they used for nutrition content, the topics they were exposed to, their perceptions of the content, for example, engagement and trust, and how certain characteristics, for example, length of content, affect their perceptions and preferences. The questions were pilot tested in a sample (n=3) of young adults, and minor changes to the wording and structure of questions were made for clarity. See Multimedia Appendix 2 for the interview guide.

Interviews were conducted by a single interviewer (BTL) via the digital video calling platform Zoom (Zoom Video Communications). No other parties besides the participant and interviewer were present in the interview. The audio from the interviews was recorded on Zoom and transcribed using Otter.ai. Transcripts were edited for correctness by the interviewer (BTL), and no participants requested to read, correct, or provide feedback on their transcripts.

An online Qualtrics demographics survey, with 7 questions, was completed by participants after the interview. Questions included student status, highest educational qualification, living arrangements, employment status, and country of birth. Age, gender, tertiary qualifications in nutrition, and diet type were asked in the interview.

### Researcher Reflexivity

The interviewer (BTL) was a male PhD candidate and qualified dietitian (MNutrDiet) with experience in conducting interviews from previous university studies. BTL acknowledges that his positionality as a professional in this area may bias the questions asked during the interviews and the analysis of data. Although his awareness of these biases is considered, his perspective may influence the results and the interpretation of the findings. Compiling a semistructured interview guide with a focus on open-ended questions and including the research team throughout the data analysis process minimizes the risk of these biases. The interviewer declares no conflicts of interest or bias that may affect the collection or reporting of results.

No relationships were established before the commencement of the study. Information about the researcher was not provided to the participants.

### Data Analysis

Descriptive statistics for sample characteristics were calculated on Microsoft Excel. Sources used for online nutrition content and nutrition topics accessed were reported narratively. Reflexive thematic analysis using the Braun and Clarke approach [23,24] was performed with NVivo 14 (Lumivero) to identify patterns and construct themes that explored perceptions and preferences of online nutrition content. An inductive approach was used to build an in-depth understanding of the topic and identify minor themes that may be missed in quantitative studies [25]. Initial codes were generated from a single coder (BTL) and then reviewed via an iterative process. In consultation with the research team, final codes were discussed and grouped into initial themes. Themes were reviewed constantly among all authors to ensure that interpretation was accurate, coherent, and addressed the research questions. The involvement of all study authors throughout the reflexive thematic analysis process prevented personal and disciplinary biases from impacting the interpretation of the findings [26]. Multimedia Appendix 3 describes how the 6 phases of reflexive thematic analysis were applied in more detail.

### Ethical Considerations

The Faculty of Health Human Ethics Advisory Group at Deakin University granted ethics approval for this study (HEAG-H 148_2023). All participants provided informed verbal consent. The study data were deidentified and anonymized. All participants who completed the interview received an Aus $30 (this equates to US $19.94, at a conversion rate of Aus $1=US $0.6647) shopping voucher.

## Results

### Participants

Data saturation occurred at the twentieth interview, and as such, 20 participants were involved in the study. A total of 24 participants were initially recruited; however, 2 did not respond to the interview request, 1 was excluded because they did not meet the inclusion criteria, and 1 withdrew from the study due to a loss of interest before booking an interview. No repeat interviews were conducted. Interviews lasted between 19 minutes and 42 minutes (mean 30.2 min, SD 5.9 min).

The mean age of the participants was 22.9 (SD 2.3) years (range 18-25 years). A total of 7 participants identified as male and 13 identified as female. Of the 17 participants who completed the demographic survey, all but one had or were studying toward a tertiary qualification. Only 1 participant had a tertiary qualification in nutrition. Table 1 summarizes the participant attributes.

**Table 1:** Participant characteristics (N=20).

Characteristic		Participants, n (%)
**Gender**		
	Men	7 (35)
	Women	13 (65)
**Continent of birth**		
	Australia	10 (59)
	Asia	5 (29)
	Europe (including Russia)	2 (12)
	Missing	—^a^
**State of residence**		
	New South Wales	10 (59)
	Victoria	6 (35)
	Western Australia	1 (6)
	Missing	—
**Living arrangement**		
	Living with parents or guardians	8 (47)
	Living with partner	5 (29)
	Living with housemates or friends	2 (12)
	Living alone	2 (12)
	Missing	—
**Education**		
	Completed secondary school	7 (41)
	Completed undergraduate degree	8 (47)
	Completed postgraduate degree	2 (12)
	Missing	—
**Student status**		
	Studying undergraduate degree	6 (35)
	Studying postgraduate degree	5 (29)
	Not studying	6 (35)
	Missing	—
**Employment status**		
	Employed full-time	5 (29)
	Employed part-time or casual	10 (59)
	Not employed	2 (12)
	Missing	—
**Diet**		
	Nonrestricted (omnivore)	13 (65)
	Vegetarian	3 (15)
	No dairy	2 (10)
	No red meat	1 (5)
	Low-carb	1 (5)

^a^Not available.

### Online Nutrition Content Use

The majority of participants viewed or sourced their online nutrition content from social media (19/20, 95%) or websites (16/20, 80%). Other sources for online nutrition content that participants used included a generative artificial intelligence (AI) model (that is, ChatGPT; 1/20, 5%), apps (1/20, 5%), eBooks (1/20, 5%), email newsletters (1/20, 5%), and podcasts (1/20, 5%).

Social media was the most popular online source used to view or engage with nutrition content. Instagram was used by most participants (16/20, 80%), while YouTube (9/20, 45%), TikTok (7/20, 35%), Facebook (6/20, 30%), and Reddit (2/20, 10%) were used by some participants. Most males used or viewed nutrition content on YouTube (6/7), whereas females mainly engaged with Instagram (13/13).

The main nutrition topic that young adults viewed on social media involved specific foods and diets. High protein, low carbohydrate diets were the most popular diet viewed by participants, while paleo, vegan, Mediterranean, keto, intermittent fasting, and other diets (eg, fruitarian and carnivore) were also viewed. Participants described engaging with content that explained the health benefits of these diets and strategies on how to follow them, for example, what specific foods or meal plans to incorporate. Particular foods, such as tofu and soy, were also discussed in terms of health benefits or consequences and ways to incorporate these foods into meals. Participants also reported viewing recipes, meal preparation or meal plan ideas, and lifestyle-related content, for example, “What I eat in a day” videos on social media. Other topics accessed by participants were curated toward their specific interests or goals. This included weight loss tips, gut health, nutrition for sports performance, and supplement use.

Internet websites were also used for nutrition content by the majority of female participants (9/13) and all male participants (n=7). Internet search engines used to access websites included Google (15/20, 75%) and Bing (1/20, 5%). Participants mainly reported accessing health-related websites such as WebMD and HealthLine for nutrition content. Other websites that were accessed included news (eg, the Australian Broadcasting Company, ABC), blogs, recipes (eg, HelloFresh), and academic websites (eg, Harvard Medical School).

Nutrition topics accessed on internet websites varied and depended on the participants’ specific interests or health and nutrition goals. Topics included gut health, protein intake, sports nutrition, and supplement use. Participants also searched about the healthiness of particular foods and ingredients that they came across (eg, whey protein), and accessed recipes, meal plans, and meal preparation ideas for healthier food ideas. Internet search engines, websites, and ChatGPT were used as tools to cross-check information from other sources, such as social media or word of mouth.

I've also [searched] ChatGPT to see if the information that is given or delivered [on] social media platforms is correct.

Participant 02, 25 years. female

### Thematic Analysis Coding Tree

**Figure 1:**
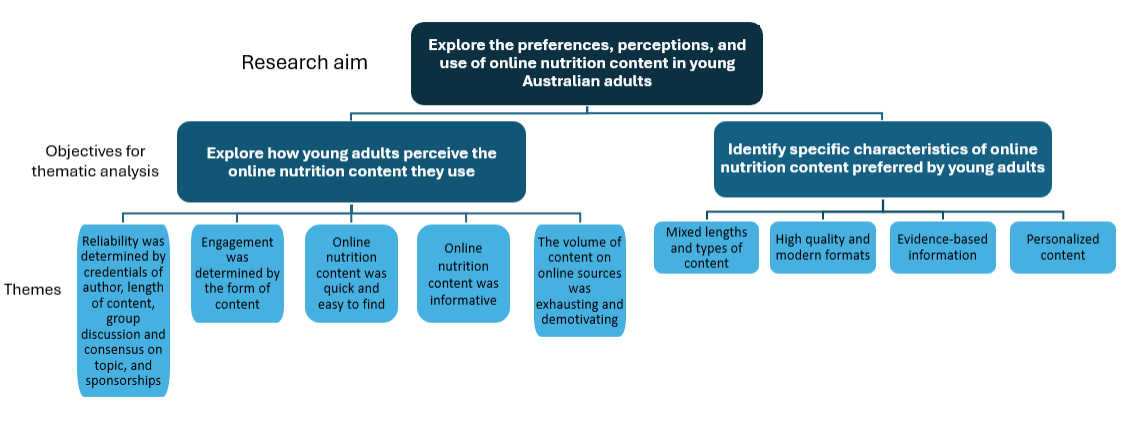
Coding tree.

A total of 5 themes were extracted to explore perceptions of online nutrition content, and 4 were extracted to identify preferred characteristics of content. Figure 1 shows the themes, which are described in detail in the following subsections.

### Perceptions of Online Nutrition Content

#### Theme 1: Reliability was Determined by Author Credentials, Content Length, Consensus on Topics, and Sponsorships

##### Subtheme 1: Authors With Professional Credentials Were Considered Reliable

Nearly all participants stated that the credentials of content creators are a main factor in determining the reliability of content. For example, those who regarded social media as reliable stated that it was because they were following or accessing nutritionists or dietitians for their nutrition content, whereas participants who regarded it as mixed or unreliable did not specifically follow health professionals and conveyed confusion in identifying and evaluating the accuracy of these credentials.

I sometimes feel that over social media, it's hard to guess if the content you're consuming [is of] high quality, [and] if the person actually knows about nutrition, or if it's just an influencer [who is] quoting what other people are saying and not based on evidence.

Participant 05, age 25 years, female

I think [a] majority of [websites are reliable] because I think [a] majority come from a specific nutrition website. If it's another website, like, for example, ABC News, giving some nutritional advice, I don't. Probably 50/50.

Participant 16, age 20 years, female

##### Subtheme 2: Short-Form Content Was Considered Less Reliable Than Long-Form Content, and Vice Versa

Nearly all participants thought that short-form content was less reliable and less trustworthy compared to long-form content.

Oftentimes, that immediate [answer] can be at the detriment to the legitimacy and quality of information you get.

Participant 07, age 24 years, female

Sometimes when it's too colourful, or too simple, I think of it as like, not reliable. So I generally go for the posts that have more information or allow me to go further to do my own research.

Participant 04, age 24 years, female

I think it's just my kind of perception [that] short form stuff being more easily […] fake.

Participant 01, age 23 years, male

Similarly, nearly all participants agreed that longer-form sources, such as websites, were more reliable than short-form content found on social media.

I think [websites are] definitely more reliable [than social media], even though it takes longer to do that type of searching […] [and] even though it's less visually entertaining. You get information through more clear and credible sources.

Participant 20, age 18 years, male

##### Subtheme 3: Discussion and Group Consensus on Topics Led to High Perceived Reliability

Only a few participants discussed the impact of comments or discussion threads and consensus between sources on perceived reliability. These participants were inclined to consider content reliable if there was an extensive discussion on the topic and if there was a consensus among those involved.

I think Reddit would be more reliable, in my opinion just because there's a whole discussion of real people.

Participant 15, age 24 years, male

I think because if so many websites are saying the same thing. Then it's like, this is agreed upon.

Participant 20, age 18 years, male

##### Subtheme 4: Sponsorships and Advertisements Led to Low Perceived Reliability

Some participants questioned the validity of content sourced from online sources as they were suspicious of funding, sponsorships, and advertisements that aimed to promote specific foods, diets, or nutritional advice.

Some podcasts that I listened to, they would specifically tell me at first: ‘this podcast is sponsored by this product or this service’. So I would keep in mind that there is some sort of influence behind it.

Participant 04, age 24 years, female

#### Theme 2: Short-Form Content Was Considered More Engaging Than Long-Form Content

Nearly all participants agreed that short-form content, prevalent on social media, was engaging. Participants credited the short lengths and dynamic formats of social media posts, for example, short videos and image posts with a description or supporting information, as key factors that led to high engagement.

It's in a visually engaging format [for] me [to] watch them through. The process that takes 30 minutes is down to a reel that lasts like 20 seconds.

Participant 11, age 25 years, female

Most participants agreed that long-form content, such as website articles, was not an engaging source for nutrition content. They expressed a lack of interest in or need to engage with sources containing long-form content, such as websites and books, which were considered visually unappealing and provided information that was hard to understand.

A lot of [website] content is just words on a page and quite descriptive, so it's not very engaging.

Participant 17, age 24 years, male

The thing is, I don't really enjoy reading. Like, I only really read if I have to. And since I'm on social media, on a daily basis, it's more accessible for me. I don't have to like search through stuff and look at [books]. [I’d] rather go on social media […] or I just search on Google.

Participant 20, age 18 years, male

#### Theme 3: Online Nutrition Content Was Quick and Easy To Find

##### Subtheme 1: Algorithms Delivered Targeted Content

Some participants credited algorithms on social media platforms for delivering content they were interested in and were more likely to engage with.

And obviously, with the algorithm, once you start looking at a few of those reels, then all your feed is about those right? So then [content] is easy to find.

Participant 11, age 25 years, female

##### Subtheme 2: Search Bars Provided Quick Answers

The majority of participants agreed that using internet search engines for obtaining nutrition content was easy to use and accessible. They reported that they were able to obtain quick answers by typing a question into a search bar.

You just have to put something into the search bar, click search, and a whole bunch of something comes out.

Participant 10, age 23 years, female

#### Theme 4: Online Nutrition Content Was Informative

##### Subtheme 1: Short-Form Formats Summarized Complex Information

Despite concerns about the reliability of short-form nutrition content, most participants regarded content as informative, helpful, and a source of inspiration (eg, recipes for meals). In addition, participants thought that content presented in an engaging manner was able to convey complex nutrition information concisely and in an easy-to-understand way.

The short-form content I access] is very short and informative, and then they tell you all the benefits of all the solutions that they're presenting.

Participant 07, age 24 years, female

##### Subtheme 2: Long-Form Formats Provided Detailed Information

Although most participants did not consider long-form content engaging, they thought that it was detailed and useful for explaining nutrition concepts.

I think most [online articles] are pretty long. Because they are long, I am not really super interested in it. So I wouldn't find myself really reading a lot. Maybe sometimes I would, but usually not. But yeah, most articles are pretty long and detailed I'd say, which is good. […] I say it's good because I feel like most people would be looking for that kind of information.

Participant 14, age 24 years, male

#### Theme 5: The Volume of Content on Online Sources Was Exhausting and Demotivating

Conflicting information, such as discussions on different diets, added to participants’ confusion. In addition, the oversaturation of content on online sources made it difficult for participants to obtain the information they needed. Some participants raised concerns regarding the pervasiveness of nutrition content and certain nutrition topics that appeared on their feed, for example, weight loss, and described the experience of using social media as exhausting and demotivating.

But I guess in the end, you just get over it. Now I just pass those videos [and] I don't give a crap. Like good on you, you lost the weight.

Participant 09, age 21 years, female

Yeah, it's a bit annoying, doom-scrolling. Sometimes it takes me a whole day, and I only find one [good recipe], and I'm like: ‘Well, I'm just buying fast food. It’s a lot easier.’

Participant 06, age 25 years, female

I try to actively avoid social media [when I want nutritional information]. But obviously, as you're scrolling, sometimes you're just inevitably given the information involuntarily

Participant 15, age 24 years, male

The majority of participants thought that the online nutrition content they accessed was overwhelming and conflicting, which demotivated them from evaluating and obtaining accurate information.

In this day and age, things are so easy to access. But then the flip side of that is people don't maintain the same value in expert information.

Participant 07, age 24 years, female

People, including myself, don't really check whether it's true or not. We're just scrolling through, and nobody's really trying to think too much.

Participant 20, age 18 years, male

So, when you go on the web, you have multiple information, right? Some people say eating veggies are good. But some people say it's bad. And some people say eating meat is good. And some people say it's not. So, it's highly subjective.

Participant 02, age 25 years, female

### Preferences for Online Nutrition Content

#### Theme 1: Short-Form Content Was Preferred

Participants provided suggestions on the format of online nutrition content to better engage and provide accurate information to young adults. Most participants desired short-form content such as Instagram reels, TikTok videos, and static image posts supported with a description or short explanation. Content that contained quick and “to the point” strategies was preferred.

I think [for] a video, I'd watch more, [compared to] reading an article or something like that.

Participant 18, age 18 years, female

I prefer like, simpler content, which is maybe more to the point or easy.

Participant 05, age 25 years, female

#### Theme 2: High-Quality and Dynamic Editing Techniques Were Preferred

Most participants preferred content that included high-quality audiovisual features and dynamic editing techniques, for example, voiceovers, text on screen, pictures or diagrams, transitions, quick video cuts, and varied camera movements, angles, and perspectives. In addition, a focus on casual and entertaining content, rather than purely educational information, was favored.

The content creators I engage with] are trying to make it entertaining instead of purely education[al]. Because […] when I'm personally watching these videos, I'm more unwinding as opposed to actively trying to learn

Participant 13, age 25 years, male

I think when [content creators] do voiceovers, it really helps. […] Also, using text on the screen helps

Participant 06, age 25 years, female

#### Theme 3: Evidence-Based Information Was Preferred

Most participants preferred content to be delivered by a health professional such as a nutritionist or dietitian. In addition, a combination of statistics and references included in nutrition content was desired.

If they give proper references, and if they give the visual statistics in front of your eyes […] it could be an easy graph, or easy bar chart, or something like that, or even an equation or a number that actually puts my head through a position where I can understand it. That's more convincing for me.

Participant 02, age 25 years, female

#### Theme 4: Personalized Content Was Preferred

Nearly all participants preferred content that was personalized to young adults. Topics of interest include high protein and bodybuilding, sports nutrition, eating for well-being, for example, gut, mental, and hormonal health. Participants also desired content that was relatable or addressed why it is important to them.

The content I access] is very targeted. It’s like: ’Oh, if you want this physique, this is how you can get it.’ And of course, I want that physique.

Participant 20, age 18 years, male

It should be informative […] and practical. Why should [I] do it? It should define the reasons and what it [means] for me.

Participant 19, age 20 years, male

## Discussion

### Principal Findings

#### Overview

This study characterized the use of online nutrition content among young Australian adults, and explored how they perceived the online sources of nutrition content they viewed or engaged with, and what preferences they had that may lead to engagement and trust. The findings suggest that young Australian adults mainly used social media, websites, and internet search engines to obtain information about nutrition; however, topics accessed differed between sources. Reported nutrition topics viewed on social media ranged considerably, whereas topics viewed from search engines and other less frequently used sources, such as eBooks, were catered toward the individuals’ interests and goals. When asked about perceptions and preferences, the interviews gravitated toward reliability and engagement of content. The young Australian adults in the study discussed the factors, such as engagement, accessibility, credentials of the author, and sponsorships, which influenced their trust in nutrition content. Content preferences were also described, that is, short formats, evidence-based information, modern features, and personalized content.

#### Perceived Reliability of Online Nutrition Content

Participants in this study stated that short-form content was less likely to be reliable compared to long-form content. On the other hand, short-form content was considered to be engaging. Short-form videos are dominant on social media due to their digestible lengths and ability to accelerate information dissemination [27]. They are highly addictive and cater to users with time constraints and shorter attention spans [28]. Although research suggests that online nutrition content on social media, particularly Instagram, is of poor accuracy and quality [29], inaccurate information is not exclusive to this platform [1]. Other frequently used sources, like commercial websites, blogs, Wikipedia, and trusted sources like government websites, may also contain inaccurate and suboptimal nutrition information [1]. Generative AI, an emerging tool for information seeking, is also known to generate inaccurate or fictional content [30], which raises concerns, as it was reported in this study to be used to fact-check content from other platforms. In addition, previous research substantiates the fact that young adults struggle to determine if information is evidence-based [10]. As such, there is a need to educate young Australian adults on evaluating the accuracy of online nutrition content on all online sources they access. This ensures they can use accurate information that may lead to positive changes to their diets and lifestyle.

In addition, the oversaturation and hyper-accessibility of content found on social media and the internet demotivates consumers from evaluating the accuracy of information accessed. Unfortunately, the accuracy of information presented online cannot be guaranteed, as misleading content can overshadow the prominence of evidence-based public health messages [1]. This is of particular concern in young Australian adults, as results indicate that they were more likely to trust content if multiple sources or authors were in agreement with each other, regardless of the content’s accuracy. Social media algorithms can further recommend inaccurate content and push users into “echo chambers,” which are defined as an environment in which extreme positions or opinions about a topic are propagated [31]. This can lead to further distrust and indifference to engage with other perspectives [32], especially from authoritative voices such as health professionals and organizations. Furthermore, this study, as well as other research [33], shows that the large volume of conflicting information accessed online can also lead to confusion and cognitive exhaustion. Over time, this can cause users to create an illusion of truth from the source [34], avoid quality information seeking [35], or become more reliant on less informed sources such as close friends and family [36].

In addition, participants who regarded internet websites or social media as reliable generally accessed content that was created by a health professional. This is consistent with previous studies, which found that consumers are more likely to trust content if it was sourced from a health website [13] or written by specific authors such as nutrition scientists and health professionals [9,13,18]. However, there is limited evidence examining whether young Australian adults actually access content created by nutrition professionals. Although accredited practicing dietitians and registered nutritionists are nutrition professionals regulated by law in Australia, other nutrition-related credentials, which are not regulated, can be misleading to lay consumers [37]. For example, degree qualifications are not required in Australia to classify oneself as a “nutritionist,” and so the credibility of content created by these authors may be varied and conflicting [38]. Therefore, differences may exist for young Australian adults in terms of perceived and actual reliability of online nutrition content. Another characteristic of online nutrition content that determined young Australian adults’ perceived reliability is external funding or sponsorships and the advertising of nutrition-related services or products. Participants expressed suspicion that content including this was less likely to be reliable. This is consistent with a similar qualitative study in young Australian women (18-35 years old; n=10), whereby participants agreed that the selling or endorsement of products led them to question the authenticity of content [39]. While this does not inherently define a piece of content to be unreliable or of low quality, provided that it is transparent and not based on testimonials, [40] it ultimately leads to lower trust and engagement in consumers.

Overall, the perceived reliability of online nutrition sources used by participants was mixed as a result of the aforementioned factors. This aligns with the actual quality and accuracy of most online sources for nutrition content, which has also been found to be mixed [1]. However, the factors that determine reliability among participants may contribute to differences in perceived and actual reliability for nutrition content. Previous research substantiates these findings as trust or confidence in nutrition content from social media and internet searches was low or mixed amongst adults [13] and university students in other countries [10,18]. A similar study in the United States also found that young adults (aged 18-25 years; n=34) thought social media posts could be misleading, or a source of inspiration, depending on the context [41]. On the other hand, young adults living in Ghana (aged 18-25 years; n=192) considered online resources as very reliable [42]. Differences may be attributed to relatively greater advancements of telecommunication infrastructure in low-income countries in recent years [42], which may lead to a more positive reception of online sources.

#### Preferences for Online Nutrition Content

Participants preferred short-form content that incorporated high-quality and dynamic characteristics that led to high engagement. This finding is consistent with a study that investigated preferences for nutrition information on Instagram in young Australian adults via an online survey (aged 18-30 years; n=108) [43]. By ranking mock Instagram posts, participants rated short video posts as more engaging and informative than posts with only text, icons, or images. They also preferred the visual style of this format and found it more likely to motivate change and present relevant information. However, in this study, participants also stated that they were less likely to trust short-form content and perceived long formats as more reliable. Therefore, it is imperative to find a balance between short, engaging formats and more detailed formats when designing online nutrition content to maintain engagement and trust with this population group. This may involve experimenting with short formats that have links or references to more detailed and evidence-based information.

Evidence-based information was also desired by participants in this study. This included the use of references and statistics in content to summarize a nutrition problem or recommendation in an easy-to-understand manner. Interestingly, this finding has not been identified previously, though it affects the quality of content [4]. A pilot audit tool found that 44% of dietitian posts on social media failed to provide references, links, or other supporting evidence [40]. As such, there is a need for health professionals to outline where information was sourced to improve the quality of content online and enable consumers to verify information.

Participants from this study also preferred casual and entertaining content, which aligns with previous research that found young adults preferred content creators who were deemed as “authentic” and “relatable” [10,39]. These studies reported that young adults were more likely to trust and engage with creators who have these qualities.

In summary, results from this study and the previous studies indicate that young adults prefer short-form content, such as short video posts, that incorporate dynamic editing techniques and casual, rather than educational content. They also desired evidence-based information in their content, such as references and statistics.

### Strengths and Limitations

A significant strength of this study was the ability to capture detailed information on young Australian adults’ use, preferences, and perceptions of online nutrition content. A qualitative approach allowed participants to openly respond to prompts, enabling a rich dataset that best captured the influences and reasons behind their use of online nutrition content. In particular, the study investigated sources and nutrition topics accessed, the reasons why they use specific sources, and why they regard them as high or low quality or accuracy. It also captured preferences from multiple digital modes and sources for nutrition content, and their characteristics, for example, length of text, supporting information like captions, use of icons and images, and links to scientific sources, which may influence their use and perceptions of particular sources.

On the other hand, sampling bias may limit the transferability of these findings to the broader young Australian adult population. First, the study included a highly educated sample, which is not representative of the young Australian adult population [44]. Highly educated individuals may be more likely to check and critique the content they access [45] and correctly identify factual information. Furthermore, a higher proportion of females than males participated in this study. Females are generally more likely to engage in protective health behaviors [46]. While efforts were made to target recruitment for males, that is, alternative recruitment materials were issued across university spaces, an overrepresentation of females was still observed. Second, information on the time spent on sources, intentionality, that is, passively viewing or intentionally searching for information, and engagement of content, that is, viewing from scrolling or accessing from searching, was not collected. Some participants in the study may have minimal engagement with online nutrition content, and as such, findings may not be transferred to highly engaged users. Future research in this area should aim to recruit a representative sample and collect relevant information, such as engagement time, when screening.

### Conclusions

This study characterized the use and identified factors that influence preferences and perceptions of online nutrition content in young Australian adults. The results can be used by health professionals in creating tailored nutrition content for this population group. The findings indicate that young Australian adults acknowledge that misinformation is not exclusive to certain platforms. The accessibility and engagement of content and the ambiguity of professional “credentials” may lead them to trust online nutrition content that is potentially of low quality and accuracy, and furthermore, disregard high-quality information. Findings also show that there needs to be a balance between short, engaging formats and presenting detailed evidence-based information when designing online nutrition content for young adults to engage these audiences, while combating nutrition misinformation. Future research in this area is needed to explore how these factors impact usage of online nutrition content and dietary behaviors, and to trial nutrition content for young adults that is both engaging and evidence-based. Further consultation with this cohort using co-design principles can help design targeted interventions to improve their food and nutrition literacy and dietary intakes.

## Appendix

Multimedia Appendix 1 Consolidated criteria for reporting qualitative studies checklist.

Multimedia Appendix 2 Interview guide.

Multimedia Appendix 3 Application of the 6 stages of reflexive thematic analysis.

## Abbreviations

AI: artificial intelligence

## Data Availability

The datasets generated or analyzed during this study are not publicly available as participants did not consent to the sharing of their data.
