# Supplementary material for: Preferences, Perceptions, and Use of Online Nutrition Content Among Young Australian Adults: Qualitative Study
Source: J Med Internet Res. 2025 Sep 29;27:e67640. doi: 10.2196/67640 (PMC12519026; doi:10.2196/67640)
Supplement: Multimedia Appendix 2 [file jmir_v27i1e67640_app2.docx]

## Introduction

Thank you for taking the time to do this interview. I really appreciate your willingness to share your experiences and insights with us.

As you may know, our study aims to understand young adults' use, perceptions and preferences of online nutrition information sources and programs.

During our time together today, we will engage in an interview that will last approximately 40 minutes. I'll be asking you a series of questions, and I encourage you to respond as openly as possible. There are no right or wrong answers – we're simply interested in your thoughts and experiences.

For this study, we will record the audio from the interview for data analysis. However, your responses will be kept strictly confidential and any personal or identifying information will be deleted after this interview. Do you consent to having the audio of this interview recorded?

Before we begin, I want to emphasise that your participation in this study is entirely voluntary. If, at any point during the interview, you feel uncomfortable or wish to stop, please let us know, and we will respect your decision without question.

## Demographic Questions

What is your age?

What is your gender identity?

Are you currently studying or have completed any nutrition qualifications?

## Use, perceptions and preferences of online nutrition content

In this section, we will ask you questions about your use and ideas regarding online nutrition content.

Where do you find or access content about nutrition?

- What websites/ apps do you access/ social media accounts do you follow?
- What nutrition messages do these websites/ apps/ social media accounts promote?
- Do you actively search for any nutrition advice or topics?

What do you think about the online nutrition content that you access?

- Do you regard this content as engaging/ reliable/ easy to use?
- What characteristics of these sources (make them/ do not make them) engaging/ reliable/ easy to use? How do you determine what information is reliable? How do you do this for content on the same platform e.g. Instagram/ Tiktok?
- How much impact do you think the nutrition content you access has on your diet?
  - Why? How?
- Why do you use online nutrition content, compared to other sources? E.g. books, newsletters, nutritionists, dietitians
  - Are there specific sources for nutrition information that you avoid? Why?

Describe what a perfect post about nutrition would look like.
