# Supplementary material for: Preferences, Perceptions, and Use of Online Nutrition Content Among Young Australian Adults: Qualitative Study
Source: J Med Internet Res. 2025 Sep 29;27:e67640. doi: 10.2196/67640 (PMC12519026; doi:10.2196/67640)
Supplement: Multimedia Appendix 3 [file jmir_v27i1e67640_app3.docx]

## Multimedia Appendix 3: Application of the six phases of reflexive thematic analysis

| Reflexive Thematic Analysis Phase | Application |
| --- | --- |
| 1: Familiarisation with the data | An initial reading of the transcripts generated by Otter.ai was conducted. After, the transcripts were re-read while listening to the corresponding audio file to gain a better contextual understanding of the data. Transcripts were edited for correctness and contextual information e.g. [sarcasm], […], [laughter], were added to the transcripts. No notes or other functions that identified or discussed data were used. |
| 2: Generating codes | Any data that addressed each of the research objectives were coded by one coder (BTL). Two separate analyses were conducted, one for each objective. The codes were constantly reviewed during repeated iterations and further familiarisation of the data. Upon review, some codes were subsumed under similar pre-existing codes if the original code had no other data items. Additionally, some codes were edited for specificity in addressing the objectives. |
| 3: Generating themes | Codes were grouped into initial themes in consultation with the research team, according to their similarity in meaning. |
| 4: Reviewing potential themes | The themes were reviewed by the research team for quality (i.e. addresses the research objectives), correctness and coherence. |
| 5: Defining and naming themes | A coding tree of the final themes were developed. Each individual theme was narratively summarised. Data items i.e. quotes, were extracted from the data to convey the diverse meanings of each theme. |
| 6: Producing the report | The write up of the analysis is provided in the analysis section. Some changes were made for correctness and clarity. At least one quote was added for each theme and/ or sub-theme to support the narrative analysis. The order of reporting for the themes was also established. |
